# The impact of tooth brushing simulation and staining thermocycling on surface roughness and color stability of CAD/CAM laminate veneers ceramic materials: an in-vitro study

**DOI:** 10.1038/s41598-026-52223-1

**Published:** 2026-05-19

**Authors:** Rasmia Salem, Reem Ashraf, Sara Elbasha

**Affiliations:** 1 Lecturer of Fixed Prosthodontics, Department of Prosthetic Dentistry, Faculty of Dentistry, King Salman International University, El Tur, South Sinai, Egypt; 2 Division of Dental Biomaterials, Department of Prosthetic Dentistry, Faculty of Dentistry, King Salman International University, El Tur, South Sinai, Egypt; 3 Lecturer of Fixed Prosthodontics, Faculty of Oral and Dental medicine, Ahram Canadian University, 6th of October City, Egypt

**Keywords:** Zirconia reinforced lithium silicate, Advanced lithium disilicate, Laminate veneer ceramic restoration, Thermocycling, Color stability, Surface roughness, Health care, Materials science, Medical research

## Abstract

This study investigated the influence of simulated brushing and staining thermocycling on surface roughness and color stability of different types of CAD/CAM laminate veneers ceramic materials. Forty-eight square-shaped specimens were created using two types of CAD/CAM glass ceramics (***ALDS***, CEREC Tessera, Dentsply Sirona, and ***ZLS***, Vita Suprinity, Vita Zahn Fabrik), each material group was divided into two subgroups (*N* = 12) according to surface finishing protocols. Surface roughness (Ra) and color parameters were measured initially, after 6 months and 1 year of brushing simulation and Coffee/Tobacco thermocycling. Data was collected and statistically analyzed using Wald-type ANOVA, and post-hoc simple effects of estimated marginal means were compared using Wald tests with Sidak adjustment. For surface roughness, all the main effects and their interactions were not statistically significant, according to ANOVA (*P* > 0.05). Color change results (∆E) showed that after one year of tooth brushing simulation and C/T thermocycling, ***Tessera finished subgroup*** had a significantly higher mean value (16.95 ± 3.06) than ***Vita Suprinity finished subgroup*** (6.67 ± 1.85) (*p* < 0.001), and the effect size was large, 0.51. While ***Tessera glazed subgroup*** had a higher non-significant mean value (7.03 ± 0.64) than ***Vita Suprinity glazed subgroup*** (6.06 ± 2.34), (*p* = 0.188), and the effect size was small, 0.02. All tested ceramics showed acceptable surface roughness values; however, tooth brushing combined with C/T thermocycling caused discoloration especially in finished Tessera CAD/CAM materials. The color change (∆E) of the two laminate veneer materials, under different surface treatments, exceeded the clinically acceptable range.

## Introduction

Prosthetic dentistry has a significant role in fulfilling the esthetic and restorative patients’ needs by introducing multiple treatment options. One of these options is Laminate veneer (LV) restorations which are considered favorable restoration in cases with high aesthetic and conservative demand. (LV) restorations showed a successful option in restoring various cases such as discolored teeth, malformed teeth and diastema closure or multiple spacing^1,2^.

Merging the (CAD/CAM) technology with modern dentistry resulted in a more precise and aesthetically pleasing dental prosthesis. CAD/CAM ceramics are characterized by their excellent biocompatibility, durability, acceptable shade, contour, and marginal adaptation compared with conventionally fabricated restorations^3^. Several monolithic ceramic blocks with various flexural strength and aesthetic properties have emerged as a laminate veneers ceramic material^4^.

One of the most popular CAD/CAM monolithic ceramic blocks is lithium disilicate glass-ceramic, manufactured first in a metasilicate phase with a flexural strength of 130 MPa and blue in color, after crystallization, these blocks become a disilicate phase with a flexural strength of 360 MPa. Moreover, lithium disilicate ceramics can be strengthened with zirconia (up to 10% by weight), this combination is said to provide the benefits of both materials. Zirconia reinforced lithium silicate glass– ceramics (***ZLS)*** has a higher flexural strength of 420 MPa and an acceptable aesthetic properties such as translucency, fluorescence, and opalescence compared to lithium disilicate ceramics^5^.

Advanced lithium disilicate (ALDS) glass ceramic is a significant advancement of glass-matrix ceramics, adding to the already known established popularity of traditional lithium disilicate materials. Unlike its predecessors, ALDS incorporates lithium aluminium silicate crystals (virgilite) into its glass enriched-zirconia matrix. This incorporation occurs during the matrix firing process, which produces new virgilite crystals and improves the material’s mechanical properties^4,6,7^.

One of the most notable characteristics of ALDS is its impressive biaxial strength, which records 700 MPa. This significant strength improvement is complemented with its superior optical and aesthetic properties, making ALDS suitable for single-unit crowns, inlays, onlays, and veneers that guarantee both durability and visually appealing characteristics. Furthermore, the manufacturer recommends fast firing process (4 min 30 s) with applying a glaze layer as a finishing step to achieve specified mechanical qualities^6,8–10^.

Regarding the long-term color stability of ceramic restorations, which is critical for achieving the best aesthetic results and patient satisfaction, and despite of the fact that the materials have improved mechanical, physical, and aesthetic properties, there are remaining concerns about veneer restorations’ color stability over time especially with smokers and high frequency of caffeine intake patients^5,11,12^.

Furthermore, Tooth brushing is also an important part of the oral hygiene measures, as tooth brushing requires mechanical action which can have significant impact on the surface roughness and gloss of ceramic restorations. These factors include abrasiveness of toothpaste, as well as the frequency and technique of brushing^13–15^.The forementioned factors may affect the color stability of these laminate veneers leading to discoloration of dental ceramic materials over time^16^.

Coffee, a popular beverage, has been known to cause discoloration due to its high pigmentation and acidity, which is exacerbated by the thermal cycling effects of hot and cold drinks, resulting in discoloration over time. Although coffee has traditionally been consumed as a hot beverage, cold coffee has become increasingly popular nowadays with the rise of diverse coffee varieties and evolving consumption cultures. Combining thermal stress (5–55 °C) with chemical staining from commonly consumed beverages may influence the discoloration behavior of veneer materials, potentially affecting their color stability and esthetic performance^17–20^.

Tobacco consumption is still widespread in today’s societies, according to the World Health Organization (WHO)^21^. Cigarette smoking is a common cause of extrinsic staining, and when paired with coffee, it may have a significant impact on the long-term esthetic results of restorations^22^.

Therefore, the combination of both tooth-brushing and thermocycling with coffee/tobacco staining solution may negatively alter the optical characteristics of ceramics, as surface roughness provides more retention areas for pigments, while higher surface free energy promotes greater adsorption of staining agents. This sequence of mechanical–thermal–chemical changes may exacerbate discoloration of ceramic materials beyond the effect of each factor separately^23–25^.

In-vitro studies that replicate real-life circumstances are critical for understanding how CAD/CAM ceramic materials perform in everyday practices. By simulating the mechanical, chemical and thermal challenges that these materials face in the oral environment, researchers can better predict their longevity and identify potential areas for improvement.

Previous studies mainly reported the effect of toothbrushing, thermocycling, coffee or tobacco staining on color stability of ceramic materials separately^4,14,18,26–28^ However, scarcity of evidence regarding the combined impact of all factors to compare finished to glazed ceramic surfaces necessitates further research rather than the individual effect of each factor solely.

Therefore, the primary objective of the study was to assess the changes in surface roughness, and color stability of these veneers’ ceramic materials with different surface treatment protocols under conditions that mimic daily oral hygiene practices and staining thermocycling.

The null hypothesis proposed that tooth-brushing simulation and coffee/tobacco thermocycling (C/T) would not produce significant changes in either surface roughness or color stability of the tested laminate veneer ceramic materials in their finished and polished, or glazed states.

## Materials and methods

**Fig. 1:**
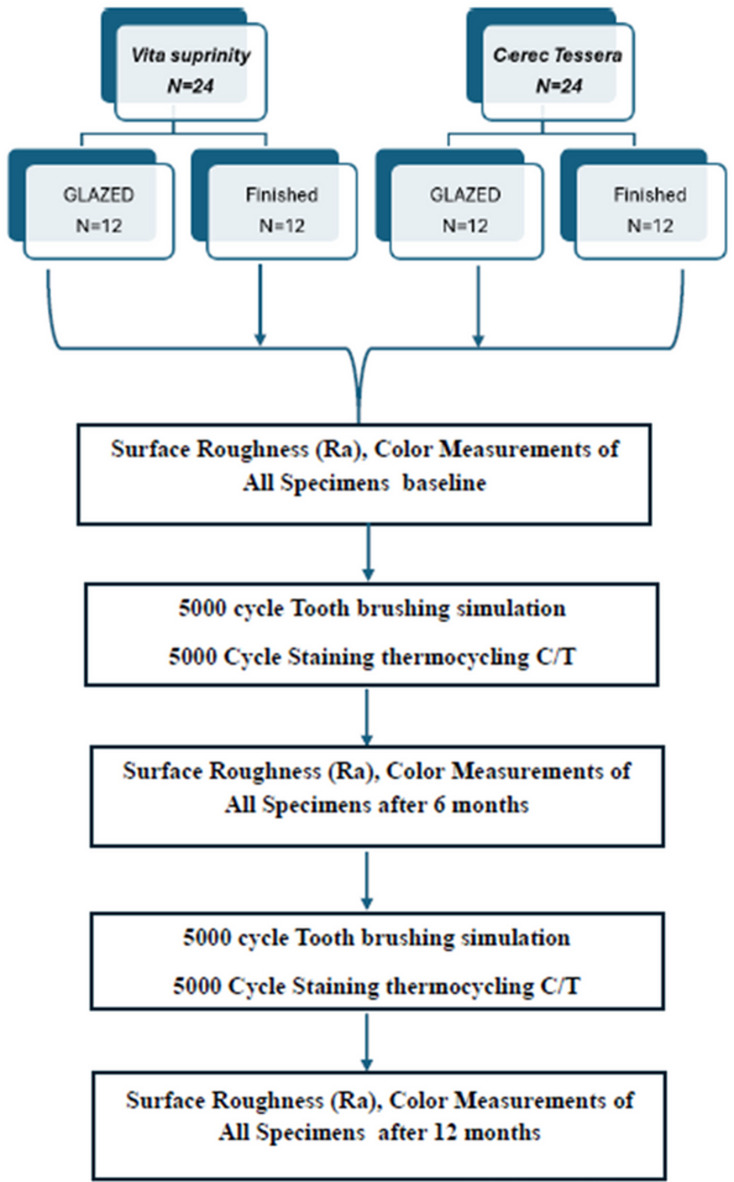
Flowchart of study design.

The Institutional Review Board Organization IORG0010868 at Ahram Canadian University’s Faculty of Oral and Dental Medicine has registered and exempted this study. Research Number: IRB00012891#126, the study design is presented in Fig. 1.

**Table 1:** Materials used.

Material	Classification	Composition	Manufacture
Tessera CT	advanced lithium disilicate glass ceramic, ALDS	Li2Si2O5: 90% Li3PO4: 5% Li0.5Al0.5Si2.5O6 (virgilite): 5%	Dentsply Sirona, York, PA, USA
Vita Suprinity, ceramic VS	Zirconia reinforced lithium silicate glass–	SiO2: 56%–64% Li2O: 15%–21% ZrO2: 8%– 12% P2O5: 3%–8% K2O: 1%–4% Al2O3: 1%–4% CeO2: 0%– 4% Pigments: 0%–4%	VITA Zahnfabrik, Bad Sackingen, Germany

### Sample preparation and grouping

This study used two types of CAD/CAM glass ceramics: Tessera **(CT)** and Vita Suprinity **(VS)** CAD/CAM blocks in shade of A2 as presented in Table 1, the flow chart of study design was illustrated in Fig. 1.

The ceramic blocks were sliced with a precision cutting machine that had adequate water cooling (Isomet 5000, Buehler, Lake Bluff, IL, USA). The samples were 6 × 6 × 0.7 ± 0.05 mm thick. All the specimens were finished on both sides under flowing water using (grit-carbide sandpapers #600, #800 and #1000 sequentially) to remove surface irregularities. After drying with air spray, All ***VS*** samples were undergoing crystallization process in a ceramic furnace (Programat EP5010, Ivoclar Vivadent) as per the manufacturer’s specifications^29^. According to **CT’s** manufacturer, the blocks comes precrystallized and no particular cycle for crystallization is necessary^4^.

The sample size was considered using G*Power (Version 3.1.6.) with a 5% alpha error and an effect size of 0.59 based on color change (ΔE)^4^.The lowest possible sample size for each test subgroup was 10 specimens, but this was raised to 12 to address possible discrepancies. 48 specimens were included in this study. The specimens were parted into two main groups based on the material, Group 1**(GPV)**: ***VS*** (*n* = 24) and Group 2 **(GPT)**: ***CT*** (*n* = 24), each group was divided into two subgroups based on the surface treatment protocol into **GPVf** (*n* = 12):***VS*** finished group, **GPVg** (*n* = 12):***VS*** glazed group, **GPTf** (*n* = 12): ***CT*** finished group, **GPTg** (*n* = 12): ***CT*** glazed group.

#### Glazed Subgroup

The samples were glazed according to the manufacturer’s instructions^4^.

#### Finished & polished Subgroup

The samples were subjected to finishing and polishing on both sides for high polished and glossy surface, the specimens were abraded lightly with a fine diamond bur using an electrical handpiece set to 10,000 rpm and in a wet environment, Optrafine F was used first to polish the ceramic specimens, followed by Optrafine P and Optrafine HP, all with a contra-angle handpiece set to 10,000 rpm, A diamond polishing paste was used with the Optrafine HP^4,14,22,29–31^.

Using a digital caliper (Absolute Digimatic; Mitutoyo, Japan), the final thicknesses of the specimens were measured. All specimens had a thickness of 0.7 ± 0.05 mm, in accordance with ISO standard 6872:2015.

### Tooth brushing simulation protocol

To simulate toothbrushing, specimens were subjected to a ROBOTA toothbrushing simulation unit (ACH-09075DC-T, AD - TECH TECHNOLOGY CO., LTD, Germany) for 5000 cycles (represent 6 months in oral cavity)^32–34^. Colgate toothpaste slurry solution was used (250 g with 1 L of distilled water) and the brush (Oral-B Leicester, UK), was replaced after 2500 cycles. To standardize the brush direction and pressure, a prefabricated holder tightened with a screw was used, and the specimens were then embedded in Teflon housing. The brush head was aligned with the bristles and had a vertical load of 2.4 N; the brushing rate was 180 strokes per minute. For removing any residual smear layer, the specimens were rinsed with distilled water for 10 min^14,15,31,35^.

### Staining thermocycling (C/T thermocycling)

The specimens were subjected to 5000 thermocycles (5–55 °C, 10 s transfer, 30 s dwell time) in hot coffee/tobacco solution and ice coffee. The staining solution was prepared as follows: the coffee brew was made with 3.4 g of coffee powder (Al Ameed Coffee Medium Turkish Coffee) and 60 ml of boiling distilled water. After 10 min, the mixture was strained through a sieve. To make the tobacco solution, 2.2 g of cigarette tobacco (rolling tobacco, captain black, USA) was mixed with 100 ml of boiling distilled water. The mixture was filtered, After 10 min, using a sieve then the filtrates were mixed to create a staining solution^36^. Cold coffee solution was prepared with cold coffee (Ice coffee, O.D. Gourmet, Amsterdam), the staining thermocycling solutions were changed every 24 h. The specimens were cleaned by brushing them ten times circumferentially with Colgate toothpaste under running water. Lastly, all the specimens were cleaned ultrasonically and dried (Jeken PS-30, Jeken, Guangzhou, China), before surface roughness and color measurements.

Then the previous procedures (tooth brushing and staining thermocycling were repeated for another 5000 cycles of tooth brushing and 5000 cycles of thermocycling) to reach total 10000 cycles representing 1 year in oral cavity)^37^.

### Surface roughness measurements

Surface roughness (Ra) was assessed by non-contact profilometer before and after the interventions (tooth brushing and staining thermocycling), all specimens were optically scanned in 3D with a high-resolution Digital camera (U500x Digital Microscope, Guangdong, China), fixed points were marked on the surface, each specimen received four measurements of its Ra values (the average (Ra) per specimen was recorded in micrometers)^15,31,38^.

### Color measurement

A reflective spectrophotometer (Model RM200QC, X-Rite, Neu-Isenburg, Germany) was used to measure the color of ceramic samples at baseline, 6 months and one year of aging (tooth brushing and staining thermocycling). To ensure accuracy, the spectrophotometer was calibrated at manufacturer-specified intervals. The samples were held in the center of a spectrophotometer’s measuring head by a white Teflon holder. This attachment was used to take multiple measurements of each specimen from the identical region, furthermore, this configuration prevented external light sources from entering the system. The size of the device’s aperture was 4 mm, and the samples were accurately aligned with the device^4,39^.

A black background was selected to make the color change value of the investigated materials more equivalent to the oral cavity^40^, the measurements were taken using the CIE L*a*b* color space relative to the CIE standard illuminant D65. The CIE L*a*b* system is a nearly uniform color space with lightness coordinates of white, black (L*), redness-green (a*), and yellowness-blueness (b*). The color difference (ΔE) between two measurements of the same specimen before and after exposure to tooth brushing and staining thermocycling is calculated using the ΔE CIELAB formula^15,16,37,40^.

$$\Delta\rm{E}_{CIELAB}=(\Delta{L}^{*2}+\Delta{a}^{*2}+\Delta{b}^{*2})^{\frac{1}{2}}$$

Where: L* = lightness (0–100), a* = (change the color of the axis red/green) and b* = (color variation axis yellow/blue).

### Statistical analysis

Continuous data was presented as means with 95% confidence intervals (CI), standard deviation (SD), minimum, and maximum values. Linear mixed models with random intercepts were initially applied to assess variable effects and interactions, with normality evaluated visually and by the Shapiro–Wilk test, and homoscedasticity by visual inspection and the Breusch–Pagan test. Both models violated homoscedasticity assumptions, and random-effects variance was near zero, indicating singular fits; thus, standard linear models were used with cluster-robust standard errors (type CR3) clustered on sample identifiers. Fixed effects were tested with Wald-type ANOVA, and post-hoc simple effects of estimated marginal means were compared using Wald tests with Sidak adjustment. Effect sizes were calculated following Cohen (1988, 1992). A significance level of *p* < 0.05 was adopted. Analysis was conducted using R version 4.5.1 (R Core Team, 2025).

## Results

### Surface roughness (µm)

**Fig. 2:**
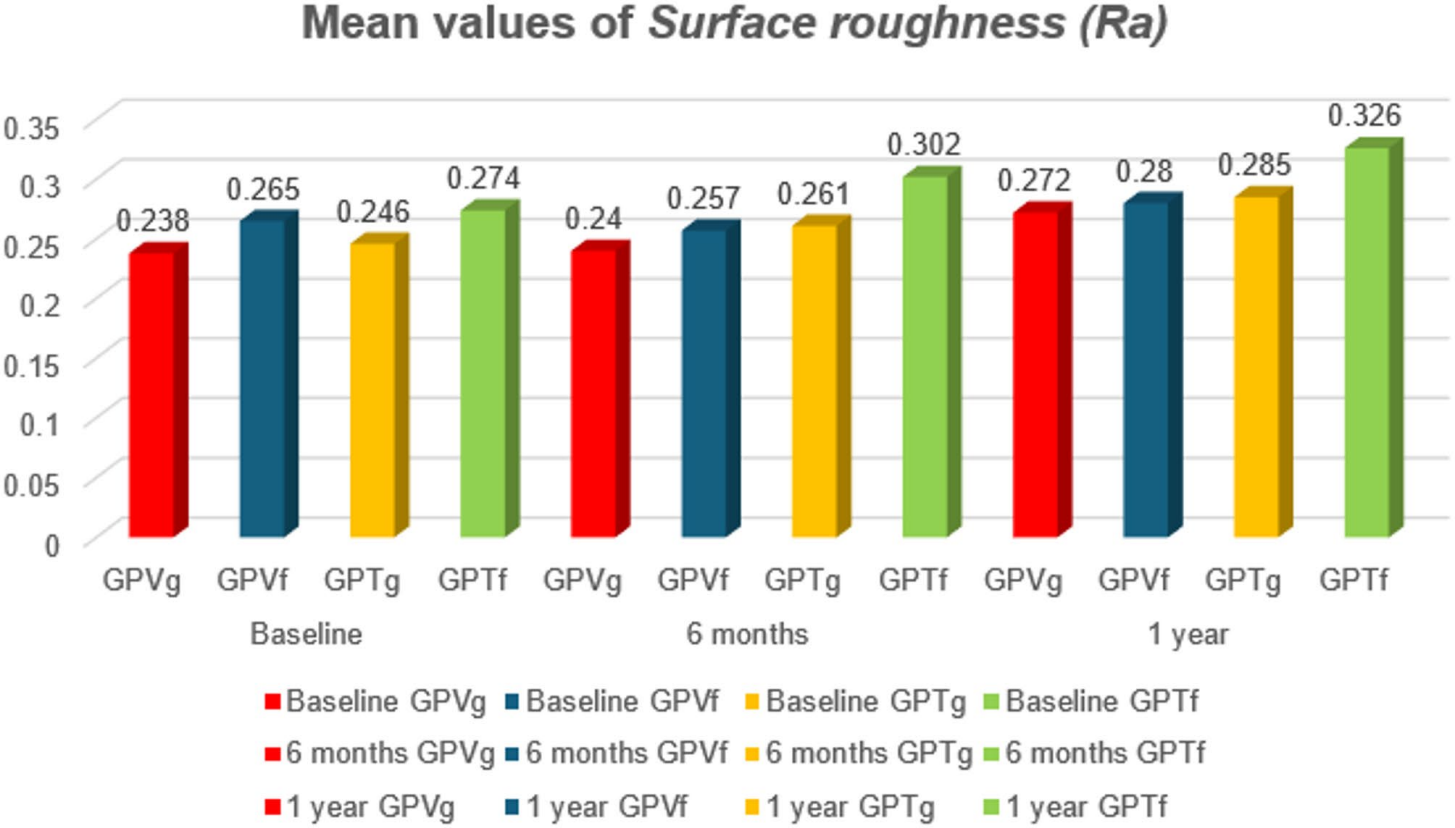
Column chart of the (Mean values) of ***Surface roughness (Ra)*** (*µ*m) for all material groups before and after tooth brushing simulation and staining thermocycling at different time intervals.

Table 2 summarizes the surface roughness (µm) results for all material groups before and after tooth brushing simulation and staining thermocycling at different time intervals, with mean values are shown graphically in Figure 2. The present descriptive statistics showed that surface roughness (Ra) of the tested ceramic materials **after 6 months and one year of tooth brushing stimulation and C/T thermocycling** : **GPTf** had the highest mean surface roughness value (0.302 ± 0.072 μm), and (0.326 ± 0.098 μm) respectively, followed by **GPTg** (0.261 ± 0.136 μm), and (0.285 ± 0.094 μm) respectively, followed by **GPVf** (0.257 ± 0.119 μm) and (0.280 ± 0.118 μm) respectively, while **GPVg** showed the lowest values (0.240 ± 0.063 μm) and (0.272 ± 0.057 μm). The difference in mean values between subgroups at different time intervals was not statistically significant, according to ANOVA test (*P* > 0.05).

**Table 2:** Surface roughness (*µ*m) results (Mean values ± SDs, 95% CI,) for all material groups before and after tooth brushing simulation and staining thermocycling at different time intervals.

Time	Material	Fi*ni*shing protocol	Mean	95% CI		SD
				Lower	Upper	
Baseline	**VS**	**Glazed (GPVg)**	0.238^A^	0.245	0.285	0.046
		**Finished (GPVf)**	0.265 ^A^	0.171	0.305	0.152
	**CT**	**Glazed (GPTg)**	0.246 ^A^	0.198	0.295	0.110
		**Finished (GPTf)**	0.274 ^A^	0.240	0.309	0.079
6 months	**VS**	**Glazed (GPVg)**	0.240 ^A^	0.212	0.268	0.063
		**Finished (GPVf)**	0.257 ^A^	0.205	0.309	0.119
	**CT**	**Glazed (GPTg)**	0.261 ^A^	0.201	0.320	0.136
		**Finished (GPTf)**	0.302 ^A^	0.270	0.334	0.072
1 year	**VS**	**Glazed (GPVg)**	0.272 ^A^	0.247	0.298	0.057
		**Finished (GPVf)**	0.280 ^A^	0.228	0.332	0.118
	**CT**	**Glazed (GPTg)**	0.285 ^A^	0.243	0.326	0.094
		**Finished (GPTf)**	0.326 ^A^	0.283	0.369	0.098

The same uppercase letter (A) denotes insignificant differences between groups. (*p* > 0.05).

### Color change (∆E)

**Fig. 3:**
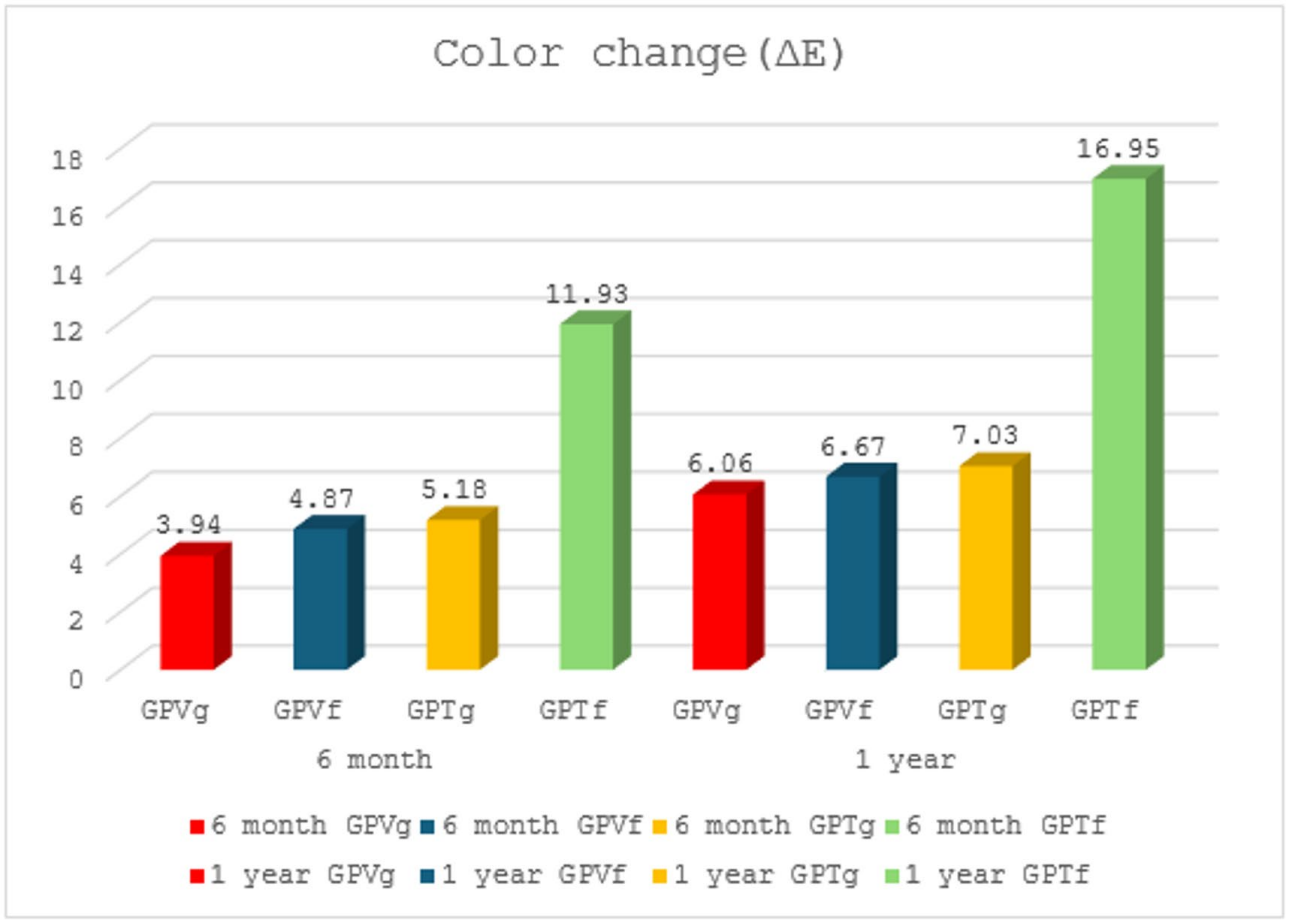
Column chart of the mean values of color change (∆E) for all material groups after tooth brushing simulation and C/T thermocycling.

Color change (∆E) results after tooth brushing simulation and C/T thermocycling at 6 months and one year, with mean and standard deviation (SD) values are shown in Table 3 and Fig. 3. the “Wald F-test” for comparing estimated marginal means showed the following:

**After 6 months: GPTf** had a significantly higher mean value (11.93 ± 1.59) than **GPVf** (4.87 ± 2.08) and the effect size was large, 0.48 (0.35 to 0.57), meanwhile, **GPTg** (5.18 ± 1.60) had insignificantly higher mean value than **GPVg** (3.94 ± 1.55) with a small effect size 0.04 (0.00 to 0.12). Table 4.

#### After one year

meanwhile **GPTf** had a significantly higher mean value (16.95 ± 3.06) than **GPVf** (6.67 ± 1.85), and the effect size was large, 0.51 (0.38 to 0.60). Meanwhile, **GPTg** had insignificant higher mean value (7.03 ± 0.64) than **GPVg** (6.06 ± 2.34), and the effect size was small, 0.02 (0.00 to 0.09).

**Table 3:** Color change (∆E) results (Mean values ± SDs) for all material groups after tooth brushing simulation and C/T thermocycling:.

Time	Finishing protocol	Color change (ΔE) (Mean ± SD)		p-value (materials)	PES (95% CI)
		VS	CT		
6 months	**Glazed**	3.94 ± 1.55	5.18 ± 1.60	0.068	0.04 (0.00 to 0.12)
	**Finished & polished**	4.87 ± 2.08	11.93 ± 1.59	< 0.001*	0.48 (0.35 to 0.57)
	**p-value**	0.233	< 0.001*		
	**PES (95% CI)**	0.02 (0.00 to 0.08)	0.53 (0.41 to 0.61)		
1 year	**Glazed**	6.06 ± 2.34	7.03 ± 0.64	0.188	0.02 (0.00 to 0.09)
	**Finished & polished**	6.67 ± 1.85	16.95 ± 3.06	< 0.001*	0.51 (0.38 to 0.60)
	**p-value**	0.505	< 0.001*		
	**PES (95% CI)**	0.01 (0.00 to 0.06)	0.56 (0.44 to 0.64)		

**Table 4:** Color change (∆E) and surface roughness among the tested materials:.

Measurement	Source	df1	df2	f-value	*p*-value	PES (95% CI)
Color change	**Material**	1	88	**163.37**	< 0.001*	**0.65 (0.55 to 0.72)**
	**Finishing protocol**	1	88	**127.93**	< 0.001*	**0.59 (0.48 to 0.67)**
	**Time**	1	88	**39.40**	< 0.001*	**0.31 (0.18 to 0.42)**
	Material * Finishing	1	88	**89.68**	< 0.001*	**0.50 (0.38 to 0.59)**
	Material * Time	1	88	**0.60**	**0.440**	**0.01 (0.00 to 0.06)**
	Finishing * Time	1	88	**3.18**	**0.078**	**0.03 (0.00 to 0.12)**
	Material * Finishing * Time	1	88	**3.97**	**0.051**	**0.04 (0.00 to 0.13)**
Surface roughness	**Material**	1	228	**0.45**	**0.502**	**0.00 (0.00 to 0.02)**
	**Finishing protocol**	1	228	**0.55**	**0.460**	**0.00 (0.00 to 0.02)**
	**Time**	2	228	**3.34**	**0.037**	**0.03 (0.00 to 0.07)**
	Material * Finishing	1	228	**1.31**	**0.253**	**0.01 (0.00 to 0.03)**
	Material * Time	2	228	**0.61**	**0.543**	**0.01 (0.00 to 0.02)**
	Finishing * Time	2	228	**0.51**	**0.604**	**0.00 (0.00 to 0.02)**
	Material * Finishing * Time	2	228	**0.13**	**0.880**	**0.00 (0.00 to 0.02)**

df: degree of freedom; PES: Partial Eta Squared; CI: Confidence interval; * significant (*p* < 0.05).

## Discussion

This study examined how surface deterioration of ceramic materials caused by tooth brushing in combination with staining solutions affects the discoloration tendency of laminate veneers ceramics materials, The coffee–tobacco mixture was selected as the staining solution due to its popularity among consumers, these products have been widely reported to cause discoloration^36^.

Color changes can be assessed traditionally or using an instrumental method. Spectrophotometer is a digital device used to measure color, minimizing human error and offering a more objective assessment of color change^40,41^.

Staining susceptibility and color changes is a crucial factor affecting the longevity of esthetic results and is influenced by several factors. Among these factors, surface roughness (R_a_) plays a prominent role, as the rough materials surface can increase the material’s susceptibility to staining especially when subjected to staining solutions and beverages over time. Higher R_a_ generally correlates with increased color instability (∆E), as higher roughness provides more surface area for stains. While surface treatments like polishing or glazing aim to reduce R_a_ and therefore, aging or staining often results in less color changes^25,42^.

Regarding the surface roughness, the null hypothesis of the current study was partially accepted, the results has shown that surface roughness (Ra) of the tested ceramic materials with various factors (finishing protocol and time intervals) did not differ significantly (*p* > 0.05) with non-significant slight increase in surface roughness of all tested groups after 1 year of tooth brushing simulation and coffee/tobacco thermocycling as assumed in the null hypothesis of the study.

The non-significant changes in surface roughness may possibly be due to the excellent physio-mechanical stability of CAD/CAM ceramics. For both ceramic materials the glaze layer improved the surface roughness in comparison to finished & polished groups which may be attributed to that the firing cycle with applying glaze layer facilitates healing of the surface cracks and pores to some extent^7^.

The present findings regarding surface roughness are consistent with those of ***Mahrous et al.***^15^ who revealed that different aging environments had no effect on pressable and CAD/CAM lithium disilicate ceramic’s surface roughness.

On the contrary, other studies found that prolonged toothbrushing cycles increased the surface roughness of the ceramic materials, The conflicting results may be due to variations in toothbrush load, type and cycles number, types of ceramic materials, and the thermocycling solution’s composition and PH^13,43^.

Regarding color change results, the null hypothesis was partially rejected. Comparisons of color change results showed that within both intervals (6 months and 1 year), there was no significant difference in the color change of glazed **VS** and **CT** samples. However, the difference was statistically significant for the finished samples, with **CT** having significantly higher change values than **VS**, and the effect size was large. Additionally, there was no significant difference between glazed and finished **VS** samples, while finished **CT** samples had significantly higher change values than glazed samples, and the effect size was large.

Thus, consumption of coffee and smoking affected the color stability of the tested ceramic materials over time, as color change (∆E) values in the tested laminate ceramics materials after staining thermocycling are not acceptable when compared to the recommended clinical references, Discoloration below Δ1.0 could be considered as not a perceptible change in color, and discolorations up to Δ3.3 could be considered as the threshold for acceptable color changes^15,36,44^.

A ceramic material’s color is governed by its chemical structure, matrix/crystals interphase change, chemical composition (crystalline content, particle size, refractive index, homogeneity, and porosity), and fabrication technique^40^,. The great color change in **CT** samples when compared with **VS** samples may be attributed to its compositional structure as it does not only contain lithium disilicate crystals but also virgilite crystals (0.5 μm), in a glassy matrix with zirconia which makes the structure less homogenous and contain more pores allowing the stain’s ingredients to invade the internal structure of the ceramic materials^14^.

On the other hand, the glazing step of **CT** samples may have been the reason the glazed samples showed lower color change in comparison to the finished samples, this step is recommended by manufacturer known as a matrix firing that alter the surface of the material eliminating cracks and creating a thin layer of nanoscale of lithium alumina silicate(virgilite crystals) boosting its final physical and mechanical properties^4,45^.

This incorporation occurs during the matrix firing process, which produces new virgilite crystals and improves the material’s mechanical properties.

Glazed porcelain generally provides a smoother surface compared to polished surface; the smooth surface may generally favor color stability (∆E*) decreasing the effect of C/T staining by time. Color stability of Tessera after glazing could be explained by ***Yot et al.***^46^, the study concluded that Tessera samples after heat treatment exhibits a more dense and less porous microstructure compared to before heat treatment, this could explain the fact that glazing step preserved the surface intact and less prone to staining.

The color change was found to be in unacceptable range after staining, this could be explained by the testing conditions, The color change after coffee/tobacco thermocycling may contribute to coffee immersion, since coffee contains approximately 22 different acids such as citric, acetic, and malic acids along with other high molecular weight acids known to contribute to discoloration, in addition surface deposition of some tobacco ingredients like cadmium, arsenic, lead, and other inorganic particles in the presence of temperature changes can cause changes of ceramic surfaces properties and which in turn affect both light transmission and reflection, also, light scattering could be affected, leading to variations in color perception^22,40,47,48^.

However, the invitro conditions of testing can be informative, but the evaluation must ultimately include measures of in-vivo conditions after cementation. Using isolated ceramic discs ignores how the veneer and cement work together once bonded, as the means of cementation is of high importance and the evaluation of its discoloration ability should be investigated.

In-vitro studies do not also give meticulous information about how salivary pellicle and bacteria affect ceramic surfaces. Staining of dental veneers can be caused by modulation of bacterial adhesion to dental biomaterials surfaces through an imbalance, or dysbiosis of the microbiota and lead to enrichment of pathobionts^49^.

Our findings align with those of ***Aydin et al.***^41^, who investigated the color changes of zirconia-reinforced lithium silicate CAD/CAM, hybrid ceramic, and composite after being immersed in popular beverages for 30 days, They reported that red wine and coffee resulted in the highest ∆E levels among the CAD/CAM ceramic materials.

Furthermore, ***Al Moaleem et al.***^45^ studied the effect of smokeless tobacco on color change of zirconia-reinforced lithium silicate ceramic (Vita Suprinity), feldspathic (Vita TriLuxe), and multilayer zirconia (Ceramill Zolid PS) restorative materials, The peak ∆E values were recorded for Vita Suprinity (4.77).

***Schelkopf et al.***^22^ also, demonstrated significant color change (*p* < 0.05) of CAD/CAM restorative materials (lithium disilicate CAD (LD), zirconia (Zr), and Telio PMMA (PMMA) in glazed (G) and polished (P) states after exposure to cigarette smoke and brushing, reporting the greatest change in mean ∆E values for glazed and polished LDS (18.62 ± 6.20), (20.26 ± 4.37) respectively.

On the contrary, ***Dederichs et al.***^36^. studied the tendency for discoloration of lithium disilicate IPS veneers and composite resin veneers after abrasion by tooth brushing simulation, abrasion and erosion, and group stored in tobacco- coffee staining mixture for twenty-one days without preceding to wear test, he found that only abrasion and erosion significantly affected the discoloration of IPS, but the results were beneath the perceptive threshold (ΔE −0.19–0.32), the presence of temperature cycles in our study may have contributed to the differences in results.

In contrast to our results, ***Demirel et al.***^4^. compared the color stability, translucency and biaxial flexure strength of Advanced lithium disilicate and zirconia reinforced lithium silicate after 6000 coffee thermocycling, the results stated that all the materials had mean ΔE00 values below the clinically perceptible threshold, indicating undetectable color changes. This controversy in the results could be due to different color measurement devices and the different composition of staining thermocycling solutions, as tobacco may have a critical influence on the color stability of different ceramic materials.

Finally, for long term color stability of ceramic restoration, the use of proper finishing and glazing protocol is of high priority, the smoother the surface, the less likely stains and bacteria would adhere to the surface of the ceramic materials. Glaze generally yields the smoothest surface, reducing stain accumulation compared to polished surfaces. Irregularities on the surface should ideally be less than 0.2 micrometer to prevent bacterial adhesion; thus, the surface roughness should be taken into consideration during clinical decision-making, emphasizing the importance of improving the properties of the material or considering alternative treatment options. In addition to the patient habits as smoking and coffee intake should be also considered during the treatment plan as they might influence the color stability of the veneers surface and would eventually discolor the ceramic surface over time^25,50^.

## Conclusion

Within the current study’s limitations, it can be concluded that:

1. All the tested ceramic materials have acceptable surface roughness values.
2. The color change (∆E) of the two tested laminate veneer materials was beyond the clinically accepted range after tooth brushing simulation, and coffee/tobacco thermocycling.
3. Laminate veneer restorations of the two tested materials with shade (A2) may not show the predictable long term esthetic success for patients with high consumption of tobacco and coffee on a daily basis.
4. Glazing of Cerrec Tessera ceramic materials may benefit in prevention of restoration discoloration by aging.
5. Careful patient counseling regarding dietary and social habits, as well as maintenance protocols aids in preserving the esthetic performance of ceramic veneers over time.

### Limitations

Corroboration with clinical studies is necessary to better understand the clinical behavior of the materials due to its different behavior in clinical circumstances as only one restoration surface is exposed to staining solutions, while the other is bonded to the tooth structure in the oral cavity, where it is exposed to different PH, salivary enzymes and microbial biofilms affecting the behavior of the materials toward staining. Moreover, using single shade (A2) may constrain the evaluation criteria of staining, as well as the small sample size, lack of specimens randomization when they were assigned to groups and the short duration of tooth brushing and thermocycling may not replicate the real lifetime restoration durability.

### Recommendation

1. Further in-vitro studies are recommended with cemented ceramic veneers to tooth structure to evaluate the roughness of the surface and color stability with large sample size and after longer duration of tooth brushing and thermocycling and different surface treatments.
2. In vivo studies are recommended to measure the color stability of the tested ceramic materials with different shades and translucencies.

## Data Availability

The data that supports the findings of this study are available on request fromthe corresponding author.

## Abbreviations

CAD/CAM: Computer aided design /computer aided manufacturing
ALDS: Advanced lithium disilicate
ZLS: Zirconia reinforced lithium silicate glass– ceramics
C/T: Coffee/Tobacco
CT: Cerec Tessera
VS: Vita Suprinity, ceramic
Ra: Surface roughness
ΔE: The color difference
